# Global gene expression under nitrogen starvation in *Xylella fastidiosa*: contribution of the σ^54 ^regulon

**DOI:** 10.1186/1471-2180-10-231

**Published:** 2010-08-28

**Authors:** José F da Silva Neto, Tie Koide, Suely L Gomes, Marilis V Marques

**Affiliations:** 1 Departamento de Microbiologia, Instituto de Ciências Biomédicas, Universidade de São Paulo, Av. Prof. Lineu Prestes 1374, 05508-000 São Paulo, SP, Brazil; 2 Departamento de Bioquímica, Instituto de Química, Universidade de São Paulo, Av. Prof. Lineu Prestes 748, 05508-000 São Paulo, SP, Brazil; 3 Departamento de Bioquímica e Imunologia, Faculdade de Medicina de Ribeirão Preto, Universidade de São Paulo, Av. dos Bandeirantes 3900, 14049-900 Ribeirão Preto, SP, Brasil

## Abstract

**Background:**

*Xylella fastidiosa*, a Gram-negative fastidious bacterium, grows in the xylem of several plants causing diseases such as citrus variegated chlorosis. As the xylem sap contains low concentrations of amino acids and other compounds, *X. fastidiosa *needs to cope with nitrogen limitation in its natural habitat.

**Results:**

In this work, we performed a whole-genome microarray analysis of the *X. fastidiosa *nitrogen starvation response. A time course experiment (2, 8 and 12 hours) of cultures grown in defined medium under nitrogen starvation revealed many differentially expressed genes, such as those related to transport, nitrogen assimilation, amino acid biosynthesis, transcriptional regulation, and many genes encoding hypothetical proteins. In addition, a decrease in the expression levels of many genes involved in carbon metabolism and energy generation pathways was also observed. Comparison of gene expression profiles between the wild type strain and the *rpoN *null mutant allowed the identification of genes directly or indirectly induced by nitrogen starvation in a σ^54^-dependent manner. A more complete picture of the σ^54 ^regulon was achieved by combining the transcriptome data with an *in silico *search for potential σ^54^-dependent promoters, using a position weight matrix approach. One of these σ^54^-predicted binding sites, located upstream of the *glnA *gene (encoding glutamine synthetase), was validated by primer extension assays, confirming that this gene has a σ^54^-dependent promoter.

**Conclusions:**

Together, these results show that nitrogen starvation causes intense changes in the *X. fastidiosa *transcriptome and some of these differentially expressed genes belong to the σ^54 ^regulon.

## Background

*Xylella fastidiosa *colonizes the xylem elements of many plants, causing diseases in economically important crops, such as citrus variegated chlorosis in citrus species and Pierce's disease in grapevines [1]. This Gram-negative fastidious bacterium, transmitted by sap-feeding insect vectors, utilizes a plethora of virulence determinants such as adhesins, type IV pili, gum and extracellular cell wall-degrading enzymes to efficiently colonize the plant xylem [2].

It has been shown that the xylem fluid affects planktonic growth, biofilm formation and aggregation of *X. fastidiosa *[3,4]. Xylem is a nutrient-poor environment that contains low concentrations of diverse compounds such as amino acids, organic acids, and inorganic nutrients. Amino acids are the main nitrogen source in xylem fluid of plants, predominantly glutamine and asparagine [5]. Recently, it was determined that glutamine predominates in the xylem sap of grapevine (*Vitis vinifera*) [3] while asparagine and glutamine are found in larger quantity in the xylem sap of citrus (*Citrus sinensis*) [6]. In infected plants, *X. fastidiosa *grows exclusively in the xylem vessels, where it must cope with nitrogen limitation and be able to utilize amino acids as nitrogen source. Although it has been determined that *X. fastidiosa *disturbs nitrogen metabolism of infected orange trees [6], no aspect of the nitrogen metabolism has been investigated in this phytopathogen.

The global response to nitrogen starvation has been studied at the transcriptional level in several bacteria, such as *Corynebacterium glutamicum *[7], *Synechocystis sp. *[8], *Prochlorococcus *[9] and *Anabaena sp. *[10]. The regulation of nitrogen metabolism is well-established in several model organisms, such as *Escherichia coli*, *Bacillus subtilis *and *Corynebacterium glutamicum *[11]. In *E. coli *and other enterobacteria, nitrogen limitation causes changes in expression of about 100 genes, whose products are involved in ammonium assimilation and scavenging for nitrogen-containing compounds [12]. Most of these genes are transcribed by the RNA polymerase containing the sigma factor RpoN (σ^54^) and activated by the nitrogen regulatory protein C (NtrC). The NtrC-RpoN regulon includes at least 14 operons, among them *glnAntrBC *(glutamine synthetase and the two-component system NtrB-NtrC), *glnK-amtB *(PII signal transduction protein and ammonium transporter), *astCADBE *(arginine catabolism), *glnHPQ *(glutamine transport) and *nac *(σ^70^-dependent transcriptional activator) [12,13]. On the other hand, in the oligotrophic alphaproteobacterium *Caulobacter crescentus *σ^54 ^does not regulate the majority of genes induced under nitrogen limitation [14].

Although the most prevalent RpoN-regulated function in bacteria is nitrogen assimilation, this alternative sigma factor controls many distinctive and unrelated cellular functions, such as pili and flagella biosynthesis, plant pathogenicity, catabolism of aromatic compounds and nitrogen fixation [15]. This is possible because σ^54 ^utilizes diverse transcription activators called enhancer-binding proteins (EBPs), all governed by their own signal pathways, for initiation of transcription [16]. Besides the absolute dependence of EBPs and ATP hydrolysis for the formation of the RNA polymerase open complex on the promoters, another unique feature of σ^54 ^is the recognition of -24/-12-type promoters with consensus sequence TGGCACG-N4-TTGC [17,18]. The σ^54 ^regulon was estimated in several organisms, such as *E. coli *[19], *Pseudomonas putida *[20] and several species of *Rhizobiaceae *[21] by use of powerful computational methods that took advantage of the high conservation of σ^54 ^promoter sequences throughout diverse bacterial groups.

Alternative sigma factors provide effective mechanisms for regulating a large numbers of genes in response to several environmental stresses. In the genome of *X. fastidiosa *there are genes encoding each of the sigma factors RpoD, RpoH, RpoE and RpoN [22]. Large-scale studies using microarrays and *in silico *analyses have permitted to determine the RpoH and RpoE regulons and their contribution to the heat shock response [23,24]. Recently, we have established that RpoN controls cell-cell aggregation and biofilm formation in *X. fastidiosa *by means of differential regulation of genes involved in type I and type IV fimbrial biogenesis. We have also characterized the first σ^54^-dependent promoter in *X. fastidiosa*, controlling expression of the *pilA1 *gene [25].

Here, we analyzed the global transcriptional profile of *X. fastidiosa *under nitrogen starvation conditions using DNA microarrays. A more complete description of the *X. fastidiosa *σ^54 ^regulon was achieved using microarray data from an *rpoN *mutant integrated with an *in silico *analysis of RpoN-binding sites. The regulatory region of the *glnA *gene that encodes the enzyme glutamine synthetase was further characterized, and confirmed to have a σ^54^-dependent promoter, suggesting an important role of ammonium assimilation mediated by σ^54 ^in *X. fastidiosa*.

## Methods

### Bacterial strains and growth conditions

The citrus strain J1a12 of *Xylella fastidiosa *[26] was cultivated in PW medium [27] without bovine serum albumin and phenol red and supplemented with 0.5% glucose (w/v) (PWG) at 25°C with no agitation. Cultures were also grown in defined XDM_2 _medium [28] or XDM_2 _medium lacking all nitrogen sources (XDM_0_) at the same conditions. For the *rpoN *mutant strain [25], 10 μg ampicillin ml^-1 ^was supplemented to the PWG medium.

### Growth of *Xylella *cells in nitrogen starvation

For time course studies, late-exponential phase cells in PWG medium were used to inoculate a culture in 100 ml XDM_2 _medium to an optical density at 600 nm (OD_600 _nm) of 0.1. Cells were grown during 12 days in the XDM_2 _medium (mid-log phase) and harvested by centrifugation. Then, the culture was divided into two portions: in one the cells were washed with XDM_2 _medium, collected by centrifugation and rapidly frozen in dry ice (this aliquot was considered the time zero of the experiment). The second portion was washed with XDM_0 _medium and the cultivation was continued for 2 h, 8 h and 12 h in XDM_0 _medium to establish nitrogen starvation conditions. For each time point, cells in a 25-ml culture were collected by centrifugation and rapidly frozen in dry ice, until RNA isolation.

### Preparation of RNA for DNA microarray

Total RNA was isolated from *X. fastidiosa *wild type and *rpoN *mutant cells, grown under nitrogen excess or nitrogen starvation conditions as described above, using the TRIZOL reagent (Invitrogen), according to the manufacturer's instructions. DNA was removed using RQ1 DNase I (Promega). RNA samples were evaluated by electrophoresis on formaldehyde-agarose gels and stored at -80°C. Microarray slides covering more than 94% of all *X. fastidiosa *genes, spotted at least in duplicate, were prepared as previously described [29]. Fluorescent-labeled cDNA preparation, microarray hybridization, washing and scanning were performed as previously described [25]. The ArrayVision version 6.0 software (Imaging Research, Inc.) was used for spot finding and signal-intensity quantification. Three RNA samples isolated from independently grown cultures of the cells at each starvation period (2 h, 8 h and 12 h) were examined, and each preparation was subjected to microarray analysis. As the genes were spotted at least in duplicate, we obtained six replicates for each gene from three independent data sets per gene per starvation period. Normalization was carried out using the LOWESS algorithm [30]. Differentially expressed genes were identified using intensity-dependent cutoff values based on self-self hybridization experiments [31]. A gene was classified as upregulated or downregulated if at least four of six replicates were outside of the intensity-dependent cutoff curves. Microarray data are available at the NCBI GEO (Gene Expression Omnibus) database http://www.ncbi.nlm.nih.gov/geo, with accession number GSE21647.

### Primer extension analysis

Primer extension assays were performed as previously described [25], using 50 μg of RNA as template isolated from J1a12 or *rpoN *cells grown in PWG. Total RNA was hybridized to the [γ-^32^P]ATP-labeled primer XF1842EXT (5'-AACAAAGCGCAAATCGACGAATTCG-3') and extended with the Superscript III reverse transcriptase (Invitrogen). The sequencing ladder was generated with the Thermo Sequenase cycle sequencing kit (USB), using the [γ-^32^P]ATP-labeled primer M13Forward (5'-GTAAAACGACGGCCAGT -3') and M13 DNA template.

### Computational prediction of σ^54^-dependent promoter sequences

A position weight-matrix was constructed using a set of 186 RpoN-dependent promoters from different bacterial species [18]. This matrix was used to perform a genome-wide screening for putative RpoN-binding sites in the *X. fastidiosa *genome sequence [22] with the PATSER module [32] from the Regulatory Sequence Analysis Tools (RSAT) website [33]. The search for putative RpoN-binding sites was restricted to intergenic regions (non-coding region between two genes) on the coding strand of all annotated genes. Sequence logos were generated using the WebLogo package [34].

## Results and Discussion

### Transcriptome of *Xylella *cells grown under nitrogen starvation

In this work, DNA microarray experiments were used to reveal the global transcriptional profile of *X. fastidiosa *under nitrogen starvation conditions. The experiments compared changes in the expression profile of cells growing in the absence of nitrogen (XDM_0 _medium) for 2, 8 and 12 hours compared to cells maintained in defined medium containing amino acids serine, methionine, asparagine and glutamine as nitrogen source (XDM_2 _medium, zero-time). The relative ratio was calculated for the zero-time sample compared with each time-point sample and data from each point correspond to three independent biological replicates. The complete list of differentially expressed genes is provided in Additional file 1: Table S1 and Additional file 2: Table S2.

**Figure 1:**
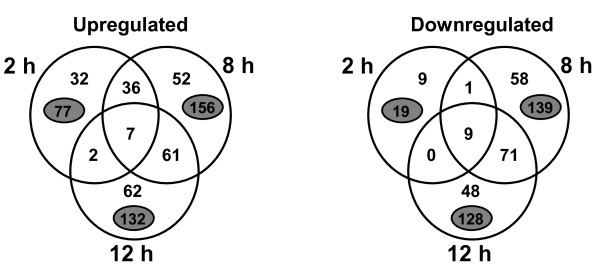
**Diagram summarizing the number of differentially expressed genes in *X. fastidiosa *J1a12 under nitrogen starvation**. Large circles represent each one of the three time-points. Numbers in the circles indicate genes with differential expression at each specific time-point and in more than one time-point (regions of intersection). Numbers in the small gray ovals indicate the total of the differentially expressed genes for each time-point (i.e. the sum of the genes in each large circle). The circles and regions of overlap are not drawn to scale.

We identified 448 differentially expressed genes at one or more time-points following nitrogen starvation and among them, 252 genes were upregulated, whereas 196 genes were downregulated (Additional file 1: Table S1 and Additional file 2: Table S2). Very few genes were up- or down-regulated during all three time-points of nitrogen starvation: 7 genes were induced and 9 genes were repressed (intersection of the three circles in Figure 1). The cumulative number of induced genes in cells exposed to 2 h, 8 h and 12 h of nitrogen starvation were 77, 156 and 132, respectively, while the number of repressed genes were 19, 139 and 128, respectively (numbers in gray ovals; Figure 1). These data indicate that the number of differentially expressed genes increased substantially from 2 h to 8 h and began to decline at the 12 h time point, indicating that the temporal series covered a wide range of genes with altered expression in response to nitrogen starvation.

The genes differentially expressed under nitrogen starvation were classified into functional classes according to the categories defined in the original annotation of the *X. fastidiosa *genome [22] based on the annotation of *E. coli *genes [35] (Table 1). There are genes belonging to all categories, but some categories are overrepresented, such as RNA metabolism (30 genes), biosynthesis of amino acids (23 genes), energy and carbon metabolism (20 genes), transport (20 genes) and protein metabolism (19 genes) (Table 1). Categories with predominance of induced genes include regulatory functions and phage-related functions and prophages. On the other hand, categories with prevalence of repressed genes compared to induced genes are mainly related to metabolism, such as central intermediary metabolism, energy metabolism and protein metabolism (Table 1). Putative functions of some of these differentially expressed genes in response to nitrogen starvation are described below.

**Table 1:** Functional classification of differentially expressed genes under nitrogen starvation in *X. fastidiosa*.

Functional Category*		**Temporal series**^**§**^	
	
	2 h	8 h	12 h
**Intermediary metabolism (25/34)**^#^			
Degradation (5/3)	2/0	1/3	2/2
Central intermediary metabolism (5/10)	4/0	2/7	3/6
Energy metabolism, carbon (3/17)	1/2	3/16	0/14
Regulatory functions (12/4)	4/1	9/2	5/2
**Biosynthesis of small molecules (28/25)**			
Amino acids biosynthesis (13/10)	9/1	8/7	3/4
Nucleotides biosynthesis (2/5)	0/0	1/2	2/5
Sugars and sugar nucleotides biosynthesis (0/1)	0/0	0/1	0/0
Cofactors, prosthetic groups, carriers biosynthesis (8/5)	2/0	6/4	2/3
Fatty acid and phosphatidic acid biosynthesis (4/4)	2/0	2/2	1/3
Polyamines biosynthesis (1/0)	0/0	0/0	1/0
**Macromolecule metabolism (28/37)**			
DNA metabolism (8/8)	1/1	5/4	7/4
RNA metabolism (17/13)	3/0	13/11	11/9
Protein metabolism (3/16)	0/6	1/15	2/13
**Cell structure (12/9)**			
Membrane components (6/3)	2/0	1/1	3/2
Murein sacculus, peptidoglycan (2/0)	1/0	0/0	1/0
Surface polysaccharides, lipopolysaccharides, and antigens (2/1)	2/0	0/1	1/0
Surface structures (2/5)	2/0	2/4	1/5
**Cellular processes (9/15)**			
Transport (8/12)	4/0	6/5	3/11
Cell division (1/3)	1/0	1/3	0/1
**Mobile genetic elements (16/7)**			
Phage-related functions and prophages (8/1)	2/0	8/1	6/0
Plasmid-related functions (7/6)	3/0	6/6	3/2
Transposon- and intron-related functions (1/0)	0/0	0/0	1/0
**Pathogenicity, virulence, and adaptation (9/13)**	1/3	6/8	5/9
**Hypothetical (122/52)**	30/5	73/34	69/31
**ORFs with undefined category (3/4)**	1/0	2/2	0/2

**Total (252/196)**	77/19	156/139	132/128

* Genes were categorized into functional classes according to the categories defined in the original annotation of the *X. fastidiosa *genome http://www.lbi.ic.unicamp.br/xf/.

^# ^The number of upregulated and downregulated genes, respectively, are indicated in parenthesis.

^§ ^Number of genes upregulated and downregulated, respectively, during time points of the nitrogen starvation temporal series.

#### Transport

Changes in expression of 20 genes encoding proteins related to transport (8 induced genes and 12 repressed genes) seem to indicate that adjustment of the transport capacity is an important cellular response to nitrogen starvation. There is a predominance of ATP-Binding Cassette (ABC) transporters, possibly involved in the transport of sugars, amino acids and iron, based on sequence annotation (Additional file 1: Table S1 and Additional file 2: Table S2). In *E. coli *[13] and *Corynebacterium glutamicum *[11] the induction of transport systems of various alternative nitrogen sources is one of the main responses to nitrogen starvation. The repression of genes encoding transporters in *X. fastidiosa *seems to be an adaptation to long time nitrogen starvation, since most of the 12 downregulated genes were repressed only at the 12 h time point (Table 1 and Additional file 2: Table S2).

#### Carbon and energy metabolism

In this category, 17 of the 20 differentially expressed genes under nitrogen starvation were repressed, most of them in the 8 h and 12 h periods (Table 1 and Additional file 2: Table S2). Genes of the major pathways of carbon and energy metabolism were repressed, including three genes of glycolysis (*pfkA*, *gapA *and *fbaB*), a gene of the enzyme pyruvate dehydrogenase (*aceE*), seven genes of the Krebs cycle (*acnB, sdhB, lpd, sucB, odhA, sucC *and *sucD*), four genes of the electron-transport chain (*etfA*, *etfB, etf-QO *and *cyoC*) and two genes of the enzyme ATP synthase (*atpA *and *atpD*). Downregulation of many genes related to carbon and energy metabolism was also observed when *X. fastidiosa *cells were exposed to prolonged high temperature [23] suggesting that this is a common response to long time stress conditions. However, genes for sugar catabolic pathways are induced by nitrogen depletion in the cyanobacterium *Synechocystis sp*. [8] and genes encoding glycolytic enzymes and respiratory chain components are upregulated during ammonium limitation in *C. glutamicum*, maybe due to the necessity of an increased ATP production during nitrogen starvation for ammonium assimilation via the GS/GOGAT pathway [36].

#### Nitrogen metabolism and biosynthesis of amino acids

After two hours of nitrogen starvation, we observed an increase in transcript levels of genes *gltD *(XF2709) and *gltB *(XF2710), encoding the two subunits of the enzyme glutamate synthase (GOGAT), while the expression levels of the *glnA *gene (XF1842), encoding the enzyme glutamine synthetase (GS), was not altered (Additional file 1: Table S1). Assimilation of ammonium by means of the high-affinity GS/GOGAT pathway is more effective than assimilation by the enzyme glutamate dehydrogenase (GDH), under nitrogen limitation. In fact, the genes encoding GS/GOGAT are upregulated under nitrogen limitation in several bacteria [12,7]. We observed induction of only few genes encoding enzymes involved in catabolism of amino acids or proteins, such as *rocF *(arginine deaminase), *tdcB *(threonine dehydratase), *pip *(proline iminopeptidase) and *pepQ *(proline dipeptidase) (Additional file 1: Table S1), suggesting that *X. fastidiosa *might scavenge nitrogen compounds as a secondary mechanism to ameliorate nitrogen starvation. The biosynthesis of amino acids was significantly affected, with 13 genes being induced and 10 genes being repressed (Table 1). However, this may reflect the fact that nitrogen starvation experiments were carried out in XMD_2 _medium, that contain amino acids (Ser, Met, Asp and Gln). The induced genes encode enzymes that are part of biosynthesis pathways of glutamate, methionine and cysteine, and their induction is probably not related to nitrogen starvation per se, but instead by the removal of these particular amino acids from the medium.

Additionally, the genes encoding RelA and SpoT, two different ppGpp synthetases that produce the nucleotide alarmone ppGpp in response to amino acids or carbon starvation [37], were induced after 2 h and 8 h of starvation. This upregulation seems to be a sign of intracellular amino acid depletion when *X. fastidiosa *cells were transferred to XDM_0 _medium. Increase in the levels of these enzymes might indicate that some functional categories containing differentially expressed genes (RNA metabolism, biosynthesis of amino acids and translation) were affected by the stringent response in addition to nitrogen starvation.

With the exception of the three genes described above (*rocF, pip *and *pepQ*), all other differentially expressed genes related to protein metabolism (16 genes) were repressed under nitrogen starvation (Table 1). Among them were genes encoding the major systems of chaperones and proteases of the cell, typical of the heat shock response, such as *groEL*, *groES*, *hspA*, *dnaJ*, *dnaK*, *grpE*, *clpB, mopA, htpX, hspA *and *mucD*, and almost all were repressed during the three time-points of nitrogen starvation (Additional file 2: Table S2). These genes are transcribed by σ^32 ^in *X. fastidiosa *[23], but the *rpoH *gene encoding σ^32 ^was two-fold induced in the 8 h and 12 h periods. This strong repression by nitrogen starvation, at least for the *groESL *operon, could be mediated by the heat-inducible transcriptional repressor HrcA, once the *hrcA *gene was four-fold induced in 2 h. Severe downregulation in the expression of genes encoding chaperones and proteases of the heat shock response by nitrogen starvation was previously observed in *E. coli *[38]. Another interesting observation was the differential expression of a large number of genes (23 induced genes and 8 repressed genes) present in the pXF51 plasmid, most of them encoding proteins of the type IV secretion system, involved in bacterial conjugation [39].

### Identifying the RpoN regulon using DNA microarrays and *in silico *analysis

In a previous work we have demonstrated, using microarray data, that few genes are downregulated in the *rpoN *mutant strain, when the experiments were performed in complex PWG medium. Under those experimental conditions, only the *pilA1 *gene (XF2542) seemed to be directly activated by σ^54^, and probably in association with the two component system PilR/PilS [25]. To determine the effect of *rpoN *inactivation on gene expression after nitrogen starvation, the transcriptomes of the wild type and the *rpoN *strains were compared using DNA microarrays, with both strains grown on XDM_2 _medium and submitted to nitrogen starvation during 2 hours. Seven of the 22 differentially expressed genes were repressed, whereas 15 were induced in the *rpoN *mutant compared to the wild-type strain (Table 2). All seven genes positively regulated by σ^54 ^were differentially expressed under nitrogen starvation (Additional file 1: Table S1 and Additional file 2: Table S2). Among them, five (XF0180, XF1121, XF1819, XF2272 and XF2542) were induced in at least one point of the temporal series (Table 2 and Additional file 1: Table S1), indicating that these genes are induced under nitrogen starvation in a σ^54^-dependent manner. Functional classification indicated four genes as related to amino acid metabolism. With the exception of the *pilA1*, which showed the highest decrease in expression in the *rpoN *mutant, all other genes were not detected in our previous microarray analysis as σ^54^-regulated genes [25]. Given that sigma factors are activators of transcription, the overexpression of 15 genes in the *rpoN *mutant compared to the wild type strain might be the consequence of secondary regulatory effects originating from the *rpoN *mutation.

**Table 2:** Differentially expressed genes under nitrogen starvation in the *rpoN *mutant compared to the wild-type strain.

Gene ID	**Product**^**§**^	**Ratio (log**_**2**_**)**^**#**^
**Downregulated genes (positively regulated by RpoN)**		
XF2542*	fimbrial protein	-3.79
XF2272*	5-methyltetrahydropteroyltriglutamate homocysteine methyltransferase	-2.21
XF1819*	threonine dehydratase catabolic	-1.62
XF1121*	5,10-methylenetetrahydrofolate reductase	-1.51
XF2699	transcription termination factor Rho	-1.37
XF0180*	hypothetical protein	-1.03
XF2207	cationic amino acid transporter	-0.80
**Upregulated genes (negatively regulated by RpoN)**		
XF1109	hypothetical protein	1.89
XF2343	recombination protein N	1.63
XF0887	mannosyltransferase	1.61
XF1830	nitrile hydratase activator	1.52
XF2551	conserved hypothetical protein	1.46
XF1658	phage-related repressor protein	1.30
XF1781	hypothetical protein	1.29
XF1117	hypothetical protein	1.24
XF2555	lysyl-tRNA synthetase	1.23
XF1469	conserved hypothetical protein	1.17
XF1078	DNA uptake protein	1.16
XF0412	nitrate ABC transporter ATP-binding protein	1.14
XF0318	NADH-ubiquinone oxidoreductase, NQO14 subunit	1.08
XF0221	hypothetical protein	0.94
XF2377	hypothetical protein	0.81

^§ ^Predicted function based on sequence similarity.

^# ^Log ratio of fluorescence intensity in strain *rpoN *compared to the J1a12 strain [log_2_(I_rpoN_/I_J1a12_)], both grown up under nitrogen starvation during two hours. Microarray analyses were carried out for three independent biological samples and a gene was classified as differentially expressed if at least four of its six replicates were outside the intensity-dependent cutoff curves.

* Genes induced under nitrogen starvation in at least one point of the temporal series.

**Figure 2:**
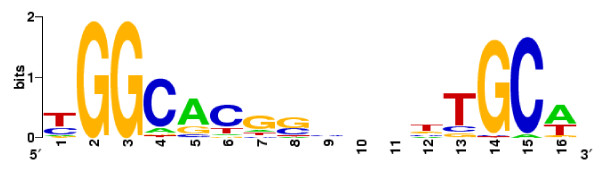
**Sequence logo for *Xylella *RpoN-binding site**. RpoN-binding sites predicted by PATSER (44 sites with score >7.95 shown in Table 3) were used to create the logo with the WebLogo generator http://weblogo.berkeley.edu/.

To potentially discriminate between genes directly and indirectly regulated by RpoN and to identify other members of the σ^54 ^regulon undetected by microarray analysis, we carried out an *in silico *search to locate potential RpoN-binding sites in *X. fastidiosa *genome. The intergenic regions of the complete genome sequence of *X. fastidiosa *were scored against a strong position-specific weight matrix derived from 186 known σ^54^-binding sites of 44 different bacterial species [18]. Considering only predicted sites with scores above the numerically calculated cutoff score (7.95), we were able to find 44 putative σ^54^-binding sites or σ^54^-dependent promoters that could potentially direct the transcription of a gene in the correct orientation. Their sequences with the associated genes or putative operons are summarized in Table 3. DNA sequence logo derived from these 44 predicted RpoN-binding sites shows two blocks of conserved sequences containing the highly frequent GG and GC dinucleotides (Figure 2), consistent with -24/-12-type promoters recognized by RpoN in most of bacterial groups [18].

**Table 3:** Predicted RpoN-binding sites in *X. fastidiosa *genome.

Gene ID	Position*	Sequence	Score	Product
XF2542	-76	TGGCACACCTTCTGCT	12.38	fimbrial protein
XF1354	-122	TGGTACGGTATTTGCT	11.58	MarR family transcriptional regulator
XF0158	-127	CGGCACGTGTGTTGCT	11.32	hypothetical protein (XF0158-59-60)
XF1842^#^	-46	TGGTATGCCAATTGCT	10.52	glutamine synthetase
XF0623	-246	TGGCACGGGAATTGAA	10.62	hypothetical protein
XF0220	-129	TGGGATGGTTCTTGCT	10.46	proline dipeptidase
XF0178	-177	TGGCATGCCAAATGCA	10.39	conserved hypothetical protein (XF0178-79)
XF0414	-189	TGGCGAGCATCTTGCA	10.29	hypothetical protein (XF0414-15)
XF1850	-7	CGGCACATGCGTTGCT	10.26	hypothetical protein (probable transposase)
XF1471	-230	CGGCACGGAATTCGCA	10.22	hypothetical protein
XF1315	-116	AGGCACTGCGGTTGCA	10.10	hypothetical protein (XF1315-*relA*-XF1317-18)
XF0746	-227	TGGCACTGCCAATGCA	9.93	hypothetical protein
XF1121	-82	CGGCACGACCCCTGCC	9.42	5,10-methylenetetrahydrofolate reductase
XF0010	-63	TGGTCCGGCCAGTGCA	9.36	biopolymer transport ExbB protein (*exbB-exbD-exbD2*-XF0013)
XF0507	-213	CGGCGCGGGTTTCGCT	9.29	hypothetical protein (XF0507-08)
XF1784	-151	TGGCACGTCAAGCGCA	9.26	hypothetical protein (ParB-like nuclease domain) (XF1784-83-82-81)
XF1943	-342	CGGCACGCTGATGGCA	9.20	histone-like protein
XF0305	-65	GGGCACCATATTTGCT	9.14	NADH dehydrogenase subunit A (*nuoABCDEFGHIJKLMN*)
XF1249	-207	CGGCCCGCAGCATGCT	8.97	hypothetical protein
XF1749	-27	TGGCGCGGCGTTTCCT	8.92	MFS transporter (XF1749-48-47-46)
XF0290	-30	CGGCACTGCCACTGCA	8.90	aconitate hydratase
XF2580	-109	CGGCACGGAGGCGGCA	8.81	30S ribosomal protein S2
XF2639	-43	TGGCGCGCCACTTTCT	8.79	preprotein translocase subunit SecE (*secE-nusG*)
XF0177	-161	TGGCCTGCATTTGGCA	8.79	hypothetical protein
XF2260	-305	TGGAACAGAAGGTGCT	8.75	alanyl dipeptidyl peptidase
XF1213	-151	CGGCTCCCCTCTTGCT	8.74	GTP-binding elongation factor protein
XF2724	-28	TGGCACAGTGCCAGCA	8.69	type I restriction-modification system (XF2724-23-22-21)
XF2677	-164	GGGCGTGATGCTTGCA	8.65	L-ascorbate oxidase
XF1609	-164	TGGCAGGTGTTGTGCT	8.60	MFS glucose/galactose transporter (XF1609-10-11)
XF2745	-15	CGGCGTGGCCGGTGCA	8.59	hypothetical protein
XF0695	-50	AGGCGCGCCGTTCGCA	8.59	hypothetical protein
XF1355	-223	TGGCAGTGCCGGTGCA	8.51	hypothetical protein
XF2501	-183	CGGCACGGAGGGGGCA	8.44	hypothetical protein (phage-related protein)
XF0710	-183	CGGCACGGAGGGGGCA	8.44	hypothetical protein (phage-related protein)
XF2093	-263	TGGCATCCAAAGTGCA	8.40	HlyD family secretion protein (XF2093-94)
XF1640	-56	TGGCAGTGCTACTGCA	8.40	ankyrin-like protein
XF2008	-44	CGGCACGCAACACGCA	8.30	hypothetical protein
XF2733	-86	TGGCAACCGCATTGCG	8.28	hypothetical protein
XF2408	-25	AGGCCCCGCAGTTGCG	8.28	hypothetical protein (XF2408-09-10)
XF0567	-16	TGGAGCACTCTTTGCA	8.22	hypothetical protein
XF2358	-36	TGGAACGCAATCTGCG	8.17	23S rRNA 5-methyluridine methyltransferase
XF0726	-255	TGGCGTGGTGGCCGCA	8.14	hypothetical protein (XF0726-27-28-29)
XF2202	-80	GGGGATGGGTGTTGCT	8.11	hypothetical protein
XF0625	-46	TGGAATTGCTATTGCT	8.11	hypothetical protein
XF0641	-179	TGGCAAAGCGGTTGAA	8.07	DNA methyltransferase (XF0641-40)

* Distance between the -12 region of the promoter relative to the initiation codon.

^# ^Predicted RpoN-binding site detected upstream of the re-annotated initiation codon of XF1842 (*glnA*).

Functional classification of the genes associated to predicted RpoN-binding sites reveals the involvement of σ^54 ^with several cellular functions, such as motility, transcription regulation, transport, carbon metabolism and protein degradation among others. However, a large number of genes (50%) encode proteins that have no attributed function (Table 3). The highest scoring RpoN-regulated promoter was located upstream of the *pilA1 *gene (XF2542), confirming a promoter previously characterized by primer extension analysis and the role of σ^54 ^in pili biogenesis [25]. The next best hit was found in front of a gene encoding a MarR transcriptional regulator (XF1354), the only regulatory gene associated with RpoN-binding site in our *in silico *analysis. MarR-like regulators control a variety of biological functions, including resistance to multiple antibiotics, organic solvents, sensing of aromatic compounds and regulation of virulence [40]. A regulatory gene belonging to σ^54 ^regulon could explain how RpoN might indirectly control the expression of genes that are not associated with RpoN-binding sites.

Predicted RpoN-binding sites were identified upstream of four putative operons encoding transport systems: two operons encoding translocases of the major facilitator superfamily (MSF) (XF1749-48-47-46 and XF1609-10-11), one operon encoding resistance-nodulation-cell division (RND) family efflux pump (XF2093-94) and the *exbB-exbD-exbD2*-XF0013 operon. Genes encoding transporters are regulated by sigma 54 in various bacteria such as *E. coli *[19], *P. putida *[20] and *Rhizobiaceae *[21], although most of these transporters are of the ATP-Binding Cassette (ABC) type. Other functional categories identified were carbon and energy metabolism (*nuo *operon encoding NADH dehydrogenase and *acnA *encoding aconitase hydratase), biosynthesis of small molecules (XF1121, XF2677 and XF1315-*relA*-XF1317-18), DNA metabolism and translation. Possible RpoN-binding sites were also found upstream of two genes encoding putative peptidases (XF0220 and XF2260). In *E. coli *the *ddpXABCDE *operon (DdpX is a D-alanyl-D-alanine dipeptidase) is induced under nitrogen limitation, possesses a potential σ^54^-dependent promoter and seems to work scavenging D-alanyl-D-alanine from peptidoglycan [13,19]. These results suggest that scavenging of nitrogen compounds could also be a mechanism controlled by σ^54 ^in *X. fastidiosa*.

To compare microarray data with *in silico *predictions, the genes and/or operons associated with the 44 predicted σ^54^-binding sites were cross-examined with the list of genes induced under nitrogen starvation (Additional file 1: Table S1) and the genes with decreased expression levels in the wild type compared to its *rpoN *derivative mutant (Table 2). Genes encoding the pilin protein of the type IV *pili *(XF2542) and methylenetetrahydrofolate reductase (XF1121), an enzyme that catalyzes the conversion of methylenetetrahydrofolate to methyltetrahydrofolate, the major methyl donor for conversion of homocysteine to methionine were induced under nitrogen starvation, downregulated in the *rpoN *mutant and were preceded by σ^54^-dependent promoters. A set of six genes possessing σ^54^-dependent promoters (XF0220, XF0308, XF0318, XF0159, XF0567 and XF1316) was induced under nitrogen starvation, but they were not differentially expressed in the *rpoN *mutant. All other genes showed no consistent correlation between the transcriptome analysis and the computational promoter prediction. These apparent divergences can be attributable to low expression of RpoN- regulated genes unless under specific conditions that activate the enhancer binding proteins, suggesting that both methods are necessary to achieve a more complete description of the *X. fastidiosa *σ^54 ^regulon. These combined strategies have been applied to determine RpoN regulon in several bacteria, such as *Listeria monocytogenes *[41], *Geobacter sulfurreducens *[42] and *Bradyrhizobium japonicum *[43].

### Detection and validation of a σ^54^-dependent promoter in the *glnA *gene

**Figure 3:**
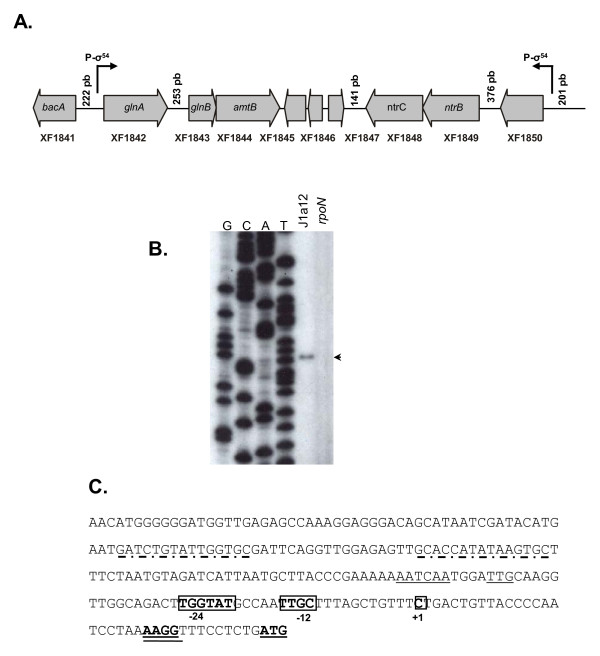
**Characterization of a σ^54^-dependent promoter in the *glnA *gene**. (A). Genomic context of *glnA *gene in the *X. fastidiosa *chromosome indicating other genes associated with nitrogen metabolism. (B). Determination of the transcription start site of *glnA *by primer extension assay. Reactions were performed using total RNA from J1a12 and *rpoN *strains and the [γ-32P]ATP-labeled primer XF1842EXT. A DNA sequencing ladder of phage M13mp18 was used as molecular size marker. The arrow indicates the band corresponding to the extended fragment. (C). Nucleotide sequence of *X. fastidiosa glnA *promoter region. The transcriptional start site determined by primer extension analysis and the -12 and -24 conserved sequence elements of the σ^54^-dependent promoter are boxed. The re-annotated initiation codon (ATG) and the putative IHF binding site are underlined. The predicted Shine-Dalgarno sequence is double underlined. The putative NtrC binding sites are indicated by dashed lines.

Analysis of genomic context indicates that *Xylella *possesses a conserved gene cluster predicted to encode proteins related to nitrogen metabolism including glutamine synthetase (XF1842), nitrogen regulatory protein P-II (XF1843), ammonium transporter (XF1844) and NtrB/NtrC two-component system (XF1848/XF1849) (Figure 3A), all genes known to be part of the NtrC-RpoN regulon in *E. coli *[13,19]. In our original analysis using the PATSER program, only one RpoN-binding site was predicted in this region. It is located upstream of the XF1850 gene that encodes a hypothetical protein containing a conserved region of a probable transposase family (Table 3). It seems unlikely that this site regulates the *ntrB-ntrC *operon, since there is a 376 bp-intergenic region between the two genes. Surprisingly, our global *in silico *prediction failed to detect RpoN-binding site upstream of the *glnA *gene (XF1842), a well-known and widespread member of the σ^54 ^regulon [19]. However, a more detailed analysis, using ClustalW alignment, indicated that XF1842 ORF was annotated incorrectly and the coding sequence should be 108 bp shorter than previously proposed. *In silico *analysis using the PATSER program in this new intergenic region detected a strong RpoN-binding site (score 10.52, Table 3).

To identify the 5' end of the *glnA *transcript, primer extension assays were performed with total RNA isolated from the wild-type and *rpoN *mutant strains. One major cDNA product was observed corresponding to a single transcriptional start site at a cytosine located 35 bp upstream of the *glnA *re-annotated initiation codon in the wild type strain, but no cDNA product was observed when primer extension experiments were performed with the *rpoN *mutant (Figure 3B). Upstream of the *glnA *transcription start site we found the predicted RpoN-binding site, a sequence (TGGTATG-N4-TTGC) that is correctly positioned and matched 9 of 11 nucleotides to the σ^54 ^consensus sequence (TGGCACG-N4-TTGC) (Figure 3C). In other bacteria, *glnA *has a σ^54^-dependent promoter and its transcription is regulated by the enhancer-binding protein NtrC [44]. Contact between the activator and the σ^54^-RNA polymerase complex is achieved by DNA looping, facilitated either by the integration host factor (IHF) protein or by intrinsic DNA topology [45]. In fact, analysis of the regulatory region of the *glnA *gene revealed the presence of AT-rich sequences with perfect match for the IHF binding site (AATCAA-N4-TTG) besides two putative NtrC-binding sites (Figure 3C).

In conclusion, primer extension data indicate that *X. fastidiosa glnA *gene has a single canonical σ^54^-dependent promoter, confirming experimentally the *in silico *prediction. The fact that sequences related to the NtrC and IHF binding sites exist at appropriate positions upstream of the *glnA *gene suggested that these factors act in concert with σ^54 ^to initiate *glnA *transcription. Therefore, ammonium assimilation is a cellular process controlled by σ^54 ^in *X. fastidiosa*, similarly to that observed in enteric bacteria [12]. Although at high concentrations ammonium is toxic to many plants [46] and the main source of nitrogen in the xylem sap are amino acids [5], studies using more precise analytical techniques have detected significant amounts of ammonium in the xylem sap, showing that root-to-shoot ammonium translocation does indeed occur in plants [47]. The ammonium translocated by xylem vessels and that derived from protein catabolism should be used as nitrogen source by *X. fastidiosa*, through its incorporation into glutamine by glutamine synthetase.

## Conclusions

In the present study, we used DNA microarrays to identify global gene expression changes during nitrogen starvation in *X. fastidiosa*. Nitrogen depletion in XDM_2_, a defined medium that contains amino acids as nitrogen source similarly to the xylem sap, resulted in major alterations in Xylella transcriptome. Changes in the expression were observed for several genes related to transport, RNA metabolism, biosynthesis of amino acids and translation, as well as a severe downregulation in the expression of genes related to heat shock response and carbon and energy metabolism. However, the function of several genes differentially expressed under nitrogen starvation remains unknown. In addition, we have also obtained a more detailed appreciation of the *X. fastidiosa *σ^54 ^regulon by combining computational prediction, microarray data and primer extension analysis. Among other cellular processes, RpoN controls pili biogenesis (*pilA*1) and ammonium assimilation (*glnA*), consistent with the fact that *X. fastidiosa *has only two EBPs proteins encoding NtrC and PilR ortologues. Experimental conditions that activate additional genes possessing true RpoN-binding sites remain to be determined.

## Authors' contributions

JFSN designed and performed the experimental work and wrote the manuscript. TK analyzed the microarray data. MVM and SLG participated in study design and coordination and helped to draft the manuscript. All authors read and approved the final manuscript.

## Supplementary Material

Additional file 1 **Table S1: Upregulated genes under nitrogen starvation in *X. fastidiosa *J1a12 strain**. The genes are ordered by the pattern of induction in the temporal series. M = log ratio of fluorescence intensity in nitrogen starvation (XDM_0_) compared to the control condition (XDM_2_). The values of M considered upregulated are highlighted in bold. Click here for file

Additional file 2 **Table S2: Downregulated genes under nitrogen starvation in *X. fastidiosa *J1a12 strain**. The genes are ordered by the pattern of repression in the temporal series. M = log ratio of fluorescence intensity in nitrogen starvation (XDM_0_) compared to the control condition (XDM_2_). The values of M considered downregulated are highlighted in bold. Click here for file
